# Assessment of farmers' knowledge, attitudes and control practices (KAP) to mitigate acaricide resistance and tick-borne diseases

**DOI:** 10.1017/S0031182024001331

**Published:** 2024-08

**Authors:** Sunita Jamra, Mukesh Shakya, Anant K. Jayraw, Vivek Agrawal, Mamta Singh, Anil Kumar Sharma, Gajendra N. Bhangale, Gaya P. Jatav, Nirmala Jamra

**Affiliations:** 1 Department of Veterinary Parasitology, College of Veterinary Science and Animal Husbandry (NDVSU), Mhow-453446, Madhya Pradesh, India; 2 Department of Veterinary Medicine, College of Veterinary Science and Animal Husbandry (NDVSU), Mhow-453446, Madhya Pradesh, India; 3 School of Agriculture, Uttaranchal University, Dehradun-248007, Uttarakhand, India; 4 Department of Veterinary Parasitology, College of Veterinary and Animal Sciences (MAFSU), Parbhani-431402, Maharashtra, India; 5 Department of Veterinary Pathology, College of Veterinary Science and Animal Husbandry (NDVSU), Mhow-453446, Madhya Pradesh, India

**Keywords:** acaricide, deltamethrin, epidemiology, fipronil, *Hyalomma anatolicum*, resistance, *Rhipicephalus microplus*

## Abstract

A knowledge, attitudes and control practices (KAP)-based study on ticks and tick-borne diseases (TTBD) and resistance development in ticks was conducted in Dhar district of Madhya Pradesh covering 200 livestock owners using a questionnaire. Based on our scoring criteria, results indicated only 25% (19.16–31.60) respondents possessing basic knowledge of TTBDs while 75% (68.40–80.84) respondents were not aware of TBDs. Due to lack of proper awareness of TTBDs, about 1.28 times more respondents (OR 95% CI 0.42–3.86) were having heavy tick infestations in their animals. However, about 36.5% (29.82–43.58) respondents showed a favourable attitude towards the adoption of different tick control practices; consequently, their animals showed low-level infestation. Amongst various feeding systems for animals, a mixed type of feeding system was mostly adopted by 57.5% respondents followed by manger system (37.5%) while grazing was the least adopted method (5%). Results indicated that the grazing animals were 6 times (OR 95% CI 2.93–12.28) more susceptible to ticks and possessed heavy tick infestation. Resistance status of collected tick isolates of *Rhipicephalus microplus* and *Hyalomma anatolicum* was assessed and revealed that both tick species were found resistant to deltamethrin. The goals of this study were to assess some of the underlying causes of ticks and TBD in livestock in Dhar district of Madhya Pradesh state using the KAP survey and resistance characterization of ticks.

## Graphical abstract

**Figure:**
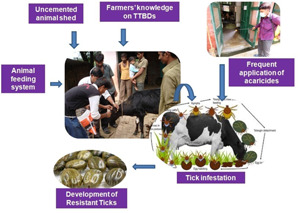

## Introduction

Tick infestation and its impact are significant challenges that limit livestock output in cattle-rearing communities mainly in tropical and subtropical countries. It is reported that about 80% of cattle population globally are adversely affected by ticks and the tick-borne diseases (TTBDs; de Castro *et al*., 1997). In India, the key species of cattle ticks are *Rhipicephalus microplus* and *Hyalomma anatolicum* (Ghosh *et al*., 2006) which serve as a vector of fatal diseases like anaplasmosis, babesiosis and theileriosis. Globally, ticks cause economic annual losses of US$22–30 billion in cattle by transmitting tick-borne diseases (TBDs) (Hurtado and Giraldo-Rios *et al*., 2018). In Brazil, *R. microplus* alone causes a loss of $32.4 million per year (Grisi *et al*., 2014). However, losses estimated due to TTBDs varies by country such as $3.0 million (Graham and Hourrigan, 1977) in the USA, $573.16 million in Mexico, $168.0 million in Colombia (Rodríguez-Vivas *et al*., 2017), $250.0 million in Australia (Meat and Livestock Australia report 2020), $364.0 million in Tanzania (Kivaria, 2006), $6.7 million in Puerto Rico and $5.0 million in Zambia (Senbill *et al*., 2018). In India, economic impact of TTBDs was estimated over $787.63 million per annum (Singh *et al*., 2022).

No specific study was focused to create a more efficient, long-lasting and comprehensive tick control approach, or to assess the performance of existing control strategies beyond the traditional application of acaricides (Jongejan and Uilenberg, 2004). In India, generally, 4 chemical classes of acaricides, i.e. organophosphates, synthetic pyrethroids, amidines and avermectins (Fular *et al*., 2021) are commonly used for tick management. Some of these chemicals are not effectively working against ticks in many parts of the country due to the development of acaricidal resistance (Bisht *et al*., 2021; Shakya *et al*., 2023). The use of acaricides on animals is prevented due to increase in resistant tick populations, their high cost, negative effects on unintended species and acaricidal residues in animal products (Singh *et al*., 2022). The success of tick control programme is based on comprehensive farmers' knowledge on TBDs, their perspective on efficient control methods and the socio-cultural environment in which the programme is carried out. The information is usually collected using the commonly used knowledge, attitude and practices (KAP) survey (Launiala, 2009). The method sets the initial standard for future evaluation and analysis of the impact of knowledge, attitude and practice on modifying TBD-related issues. It proposes an intervention approach that takes into account the unique local conditions and the cultural variables that shape them, and designs activities that are appropriate for the particular community concerned (Gumicio *et al*., 2011). Despite criticism for generalized data of a large population for planning purposes, KAP surveys on TBDs have played a significant role in developing effective intervention strategies (Butler *et al*., 2016; Zoldi *et al*., 2017; Niesobecki *et al*., 2019; Gupta *et al*., 2021).

The animal owners of Dhar district of Madhya Pradesh face problem of resistance development in ticks and their management due to lack of KAP-based data. These data are essential to formulate suitable strategy to manage resistant ticks and to improve livestock health and the income of the marginal animal owners. Thus, to tackle the problem in the targeted region, a KAP-based study was conducted to assess the influence of TBDs on livestock productivity and determination of resistance status of tick populations, and the control strategies adopted by livestock owners. The collected data will aid in creating efficient animal health initiatives to boost livestock output and to enhance the socioeconomic status of livestock owners of targeted region.

## Materials and methods

### Study area

Dhar district is located in Malwa region of Madhya Pradesh of India and was selected for conducting the KAP survey. It possesses a diverse terrain with altitudes ranging from 150 to 600 m above sea level, influenced by the Vindhya Range. The vegetation consists of dry deciduous forests, including teak, sal and bamboo, with more dense forest cover in the hilly regions. The semi-arid climate and topographical variations contribute to the presence of grasslands and scrub forests in lower areas. The geographic locations of different sub-divisions of Dhar district are Dhar (DHA, 75.32°N, 22.61°E), Manawar (MAN, 75.08°N, 22.23°E), Sardarpur (SAR, 74.97°N, 22.65°E), Kukshi (KUK, 74.75°N, 22.20°E) and Gandhwani (GAN, 75.08°N, 22.23°E). Cattle and buffaloes are primarily reared for milk production, contributing significantly to the livelihoods of the local population. However, challenges such as limited access to quality feed, veterinary services and water resources can affect the productivity of milch animals in the region. The organized farms included more than minimum 10 milch animals and well-maintained shelter with proper cemented flooring infrastructure for animals. On the other hand, unorganized farm included household animals which had mud flooring and no proper amenities and only 2 or 3 animals were maintained for personal purpose.

### Questionnaire survey

A systematic questionnaire was designed to gather data on several aspects associated with cattle productivity and TTBDs. A questionnaire proforma was designed in a multiple-choice form as per the guidelines (Thrusfield, 2018), with modifications made *via* both informal and formal testing processes. The questionnaire proforma contained several subjects like socio-demographic information, animal sheds, animal feeding methods, shed conditions, farming practices, methods of acaricidal application, risk factors, etc. The survey was carried out bi-monthly from February 2022 to January 2023 to monitor seasonal variations in cattle productivity and the prevalence of TBDs and the questionnaire was provided to livestock owners at the surveyed places. The study authors conducted the survey through face-to-face interviews with the owner. There were no specific inclusion and exclusion criteria for the participants in the study. The survey was intended for household heads; however, if there were other persons involved in livestock rearing in the household, they were asked to be in the survey as well. The questionnaire underwent pilot testing to ensure its effectiveness in gathering correct information (Williams, 2003). Prior to providing the questionnaire to the targeted participants, it was reviewed by a number of experienced investigators of epidemiological study. Then, the data were carefully collected, analysed and screened for accuracy. The farmers selected for the investigation were chosen for their willingness to participate and operational convenience. They were owners of ruminant herds consisting of 5–15 animals (Hussain *et al*., 2021). About 200 individual interviews were conducted with livestock owners using a developed questionnaire.

### Tick collection and processing

The biological samples of *R. microplus* ticks were collected from different regions of Dhar district following a randomized sampling procedure. Tick samples were collected from cattle and buffaloes of the households and well-managed dairy farms. Engorged female ticks were collected in labelled sample bottles covered with cotton cloth, and brought to the research centre. In the laboratory, at least 100–150 engorged female ticks collected from each sub-division were pooled and placed in Petri plates (5 ticks per plate) and maintained in the laboratory (Ghosh and Azhahianambi, 2007). They were then kept at 28°C and 85 ± 5% relative humidity for normal oviposition. The ticks procured from Dhar, Manawar, Sardarpur, Kukshi and Gandhwani sub-divisions were encoded as DHA, MAN, SAR, KUK and GAN isolates, respectively. The eggs laid by the female ticks from each sub-division were combined and identified as a representative sample of that sub-division. The eggs of each sub-division were pooled, collected and stored in tick-rearing tubes. Once hatched, the larvae were placed in an incubator set at 28°C and 85 ± 5% relative humidity for 8–10 days for larval-based experiments.

### Identification of ticks

The collected tick samples of both the sexes were observed morphologically under stereomicroscope. The specific characters of *R. microplus* and *H. anatolicum* were identified with the help of the book ‘*Helminths, Arthropods and Protozoa of Domesticated Animals*’ (Soulsby, 1982) and then characterized them.

### Reference tick

The reference susceptible IVRI-I strain of *R. microplus* was used as the reference tick for resistance characterization. The IVRI-I strain is already characterized as susceptible to most of the chemical acaricides in the Entomology laboratory of Indian Veterinary Research Institute, Izatnagar.

### Chemical acaricides

Technical grade deltamethrin (DLM) and fipronil (FIP) were procured from Sigma Aldrich (St. Louis, MO, USA) and their stock solutions of 5000 and 1000 ppm, respectively, were prepared in methanol. Working concentrations of DLM (60, 90, 120, 150 and 180 ppm) and FIP (10, 15, 20, 25 and 30 ppm) were prepared in distilled water from their stock solutions and were tested for resistance characterization in collected tick samples.

### Resistance characterization

#### Larval packet test (LPT)

A modified version of the larval packet test (LPT) as recommended by the FAO (2004) was used. The packets were prepared in triangular shape from Whatmann filter paper No. 1 measuring 5.5 cm × 5 cm. These packets were soaked with 0.7 mL solution of acaricide and then dried at 37°C in hot air oven. After drying, 1 side of the packets was sealed with adhesive tape. Then, about 150–200 larvae of 7–10 days old were introduced in the packets and sealed using a ‘bulldog’ clips. The packets were then kept in for 24 h in a biological oxygen demand (BOD) chamber at 28°C and 85 ± 5% relative humidity. After 24 h, these packets were removed from the BOD, and opened on white paper sheet under electric lamp to observe dead and alive larvae. The larvae only moving their legs were considered as dead while running larvae were counted as alive. Accordingly, the mortality percentage of larvae was determined by counting the number of alive and dead larvae. Three replications were maintained for each concentration of acaricides along with control with distilled water.

#### Larval immersion test (LIT)

Shaw (1966) was the initial developer of the larval immersion test (LIT). The Shaw's immersion sandwich method involves larval immersion in an acaricide solution or suspension. For the assay, more than 300 larvae were transferred into 1.5 mL microcentrifuge tubes (3 repetitions per dilution) with the help of drawing brush and then an amount of 0.75 mL of working solution of the acaricide was poured in these tubes. The larvae were submerged for 10 min and agitated intermittently. After opening the tubes, approximately 100 larvae were transferred to filter paper packets and sealed with ‘bulldog’ clips. The packets were kept at 28°C and a relative humidity of 85 ± 5% for 24 h. Control groups of each acaricide were also immersed in distilled water in the same way. After 24 h, larval mortality was assessed as mentioned in LPT.

The resistance status of field isolates was determined on the basis of resistance ratio (RR). The resistance ratio (RR50) is the ratio of LC50 value of an acaricide for field ticks and LC50 value of the acaricide for reference susceptible IVRI-I strain (Castro-Janer *et al*., 2009). Ticks were then classified according to various resistance levels as per the method of Sharma *et al*. (2012).

### Statistical analysis

The questionnaire data from 200 respondents were transferred to the Microsoft Excel 2010 sheet for proper management and analysis. The proportions of variables recorded in questionnaires were analysed following descriptive statistics (frequencies and percentages). The data were analysed by Epi Info™ software (Centers for Disease Control and Prevention, Atlanta, GA, USA). Association of socio-demographic characteristics to level of tick infestation was analysed by *χ*^2^ test. Simple logistic regression analysis through R-software package (dplyr) was also performed to observe the association of respondent's knowledge and level of tick infestations (Wickham *et al*., 2021). The dose–response data of LPT and LIT were subjected to probit analysis (Finney, 1971) using GraphPad Prism v.5 statistical software (GraphPad Software, San Diego, CA, USA) to determine LC50 values of each acaricide.

## Results

### Collection of ticks and farm management practices

The tick isolates were collected from the households and well-managed dairy farms located in 5 sub-divisions of Dhar district of Madhya Pradesh. There were both cross-bred and native breeds of cattle and buffaloes in the district. The surveyed animals were found to have a moderate (>50–100 ticksper animal) to high (>150–200 ticks per animal) level of tick infestations. Despite repeated applications of different synthetic acaricides such as cypermethrin, DLM, ivermectin and amitraz, a significant number of farmers reported the failure of tick control. During the survey, it was noticed that the application of FIP in the dairy farms is not frequent and almost lacking. However, the application schedule for other synthetic acaricides was not properly maintained and the animals were treated whenever tick infestation was visible on animals. The frequency of acaricidal treatment of household animals was comparatively lower than those maintained in well-managed dairy farms. The targeted area was highly dominated by the tribal population where animals were kept in small to big huts made of mud, concrete and thatched roofs with no proper acaricidal dose and application. It was noticed that the farmers rarely applied insecticides in the animal sheds to eradicate off the host tick stages (Table 1).

**Table 1.** Questionnaire data collected from surveyed places for determination of pattern of acaricidal application in fields

Blocks	Farm type	No. of farms visited	Commonly used acaricides	Mode of application	Frequency of application^a^	Shed treatment
DHR	Unorganized, organized	40	Deltamethrin, cypermethrin, amitraz, ivermectin	Pour on/injection/swabs	Occasional, frequent	Rarely
GAN	Unorganized, organized	40	Deltamethrin, cypermethrin, ivermectin	Pour on/injection	Occasional	Rarely
KUK	Unorganized	40	Deltamethrin, cypermethrin, ivermectin	Pour on/injection/swabs	Occasional, frequent	Rarely
MAN	Unorganized, organized	40	Deltamethrin, cypermethrin, ivermectin	Pour on/injection/swabs	Frequent	Rarely
SAR	Unorganized, organized	40	Deltamethrin, cypermethrin, amitraz, ivermectin	Pour on/injection/swabs	Frequent	Rarely

a Application frequency: frequent = 10–14 applications/tick active season; occasional = 4–8 application/tick active season.

### Analysis of socio-demographic characteristics of the respondents

The study comprised 200 farmers including literate and illiterate from 5 sub-divisions of Dhar district and more than 90% famers belonged to rural areas. It was interesting to mention that farmers showed their interest to adopt new techniques and technologies for the management of TBDs. Face-to-face interviews revealed that about 40% (33.15–47.15) respondents were literate and 60% (52.85–66.85) were illiterate, out of which 55% (47.82–62.02) were using uncemented floors and 45% (37.98–52.18) were using cemented floors for their animals. Respondents adopted different feeding methods for their animals as observed during the survey. It was found that about 37.5% (31.25–45.11) respondents fed their animals in manger, only 5% (2.42–9.00) adopted grazing system and the rest (42.5%) adopted a mixed type of feeding system. Only 25% (19.16–31.60) respondents had knowledge about TTBDs while 75% (68.40–80.84) were not aware of it. During the study, we observed that the cypermethrin was preferred for animal application by the livestock owners [i.e. 35% (28.41–42.05)] followed by DLM [29% (22.82–35.82)] and ivermectin [15% (10.35–20.72)] while only 9% (5.42–13.85) farmers were applying amitraz for tick control. The respondents [12% (7.84–17.33)] were using more than 1 acaricide without maintaining any fixed application pattern and hence they were considered in the mixed category. A favourable attitude towards different tick control methods was shown by 36.5% (29.82–43.58) respondents in which manual hand picking as well as chemical control methods were the most preferred methods (Table 2).

**Table 2.** Socio-demographic characteristics of the respondents

Variables	Categories	Frequencies	Percentage (95% CL)
Gender	Male	126	63.00 (55.91–69.70)
	Female	74	37.00 (33.30–44.09)
Education	Literate	80	40.00 (33.15–47.15)
	Illiterate	120	60.00 (52.85–66.85)
Type of housing	Kachha floor	110	55.00 (47.82–62.02)
	Pakka floor	90	45.00 (37.98–52.18)
Feeding system	Grazing	10	05.00 (2.42–9.00)
	Manger	75	37.50 (31.25–45.11)
	Mixed	115	57.50 (49.83–63.96)
Knowledge about TTBDs	Yes	50	25.00 (19.16–31.60)
	No	150	75.00 (68.40–80.84)
Attitude towards tick control	Favourable	73	36.50 (29.82–43.58)
	Unfavourable	127	63.50 (56.42–70.18)
Commonly used acaricides	Cypermethrin	70	35.00 (28.41–42.05)
	Deltamethrin	58	29.00 (22.82–35.82)
	Ivermectin	30	15.00 (10.35–20.72)
	Amitraz	18	09.00 (5.42–13.85)
	Mixed	24	12.00 (7.84–17.33)

### Association of socio-demographic characteristics to level of tick infestation by *χ*^2^ test

Analysis by *χ*^2^ test revealed that the animals of literate respondents were significantly (*P* < 0.05) less susceptible to tick infestation as compared to those of illiterate respondents (*P* = 0.0180). The animals kept in the uncemented floor of shed exhibited a high intensity of tick infestation (0.0029). The animals having manger feeding were observed to be less susceptible to ticks as compared to grazing and mixed feeding animals (*P* < 0.0335) indicating the significant association (*P* < 0.05). Amongst 200 respondents, only 50 exhibited knowledge regarding TBDs. The data obtained were found to be statistically significant at a 5% level (*P* = 0.0063) (Table 3). Insignificant differences were observed between tick infestation level and acaricides used for tick control by respondents.

**Table 3.** Association between socio-demographic characteristics of the respondents (*n* = 200) and tick infestation

Variables	Categories	Respondents	Tick infestation level		*P* value
			High	Low	
Education	Literate	80	15	65	0.0180*
	Illiterate	120	45	75	
Shed floor type	Kachha	110	47	63	0.0029**
	Pakka	90	18	72	
Feeding system	Grazing	10	6	4	0.0335*
	Manger	75	13	62	
	Mixed	115	43	72	
Knowledge about TTBDs	Yes	50	15	35	0.0063**
	No	150	84	66	
Commonly used acaricides	Cypermethrin	70	29	41	–
	Deltamethrin	58	23	35	
	Ivermectin	30	11	19	
	Amitraz	18	7	11	
	Mixed	24	05	19	

* Significant at *P* < 0.05 **Significant at *P* < 0.01

### Association of socio-demographic characteristics to level of tick infestation by logistic regression analysis

The data analysed by logistic regression revealed that the animals of female livestock owners were 2.20 times (OR 95% CI 1.13–4.27) more likely than male livestock owners to experience tick infestation. Although the level of tick infestation was not considerably impacted by respondents' educational levels, their attitudes towards various tick-control strategies were greatly influenced. Similarly, the livestock owners without having knowledge of TBDs were 1.28 times (OR 95% CI 0.42–3.86) more likely to have a high level of tick infestation (Table 4). Moreover, the low-level infestation was recorded in the animals of respondents having a favourable attitude towards different tick control methods (OR 1.04, 95% CI 0.4–2.66). The respondents who practiced grazing as a sole method of feeding for their animals were likely to be more susceptible by 6 times (OR 95% CI 2.93–12.28) to ticks and had a heavy tick infestation as compared to mangers and mixed feeding practices. No significant difference between level of tick infestation and acaricides used for tick control was observed (Table 5).

**Table 4.** Simple logistic regression analysis for the estimation of the association between socio-demographic variables of respondents and binary outcome

Variable	Category		Estimated ± s.e.	Adjusted OR (95% CI)	*P* values
Sex	Female	Male	0.7909 ± 0.33	2.20 (1.13–4.27)	0.0191
	74	126			
Education	Literate	Illiterate	−1.074 ± 0.56	0.34 (0.11–1.04)	0.0592
	80	120			
Type of animal shed floor	Kachha	Pakka	−1.267 ± 0.36	0.28 (0.13–0.57)	0.0005
	110	90			
Knowledge about TTBDs	Yes	No	0.2527 ± 0.56	1.28 (0.42–3.86)	0.6525
	50	150			
Attitude towards tick control	Favourable	Unfavourable	0.0459 ± 0.47	1.04 (0.4–2.66)	0.9231
	73	127			
Constant	–	–	0.0289 ± 0.26	–	0.9134

Significant at *P* < 0.05; OR, odd ratio.

**Table 5.** Multiple logistic regression analysis for the estimation of the association between practices of respondents with level of tick infestation

Variable	Category	Respondents	Estimated ± s.e.	Adjusted OR (95% CI)	*P* values
Feeding	Grazing	10	1.7931 ± 0.36	6.00 (2.93–12.28)	0.0000
	Manger	75			
	Mixed	115			
Acaricides used	Cypermethrin	70	0.0194 ± 0.13	1.01 (0.78–1.32)	0.8845
	Deltamethrin	58			
	Ivermectin	30			
	Amitraz	18			
	Mixed	24			
Constant	–	–	−3.3805 ± 0.72	–	0.0000

Significant at *P* < 0.05

### Association of socio-demographic characteristics to level of tick infestation by R software analysis

**Figure 1.**
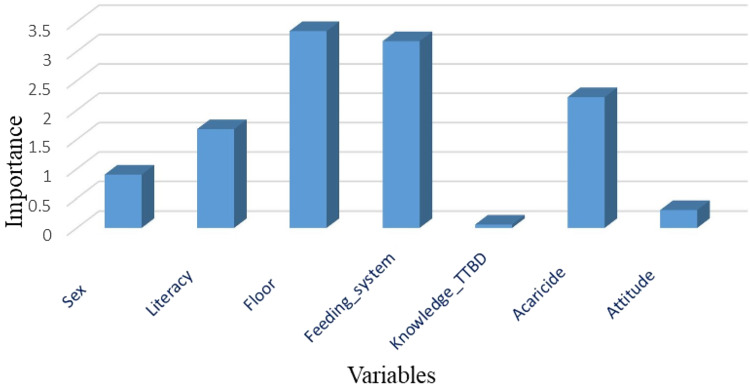
Variable importance model for each contributing factor in tick infestation.

The multiple logistic regression analysis showed that shed floor type, feeding system and acaricides used for tick control were the significant variables in this model. Respondents having uncemented animal sheds were 5.16 times (OR 5.16) more likely to have a high level of tick infestation in their animals as compared to those having cemented floor of sheds. The respondents adopted 3 types of feeding systems: grazing, manger and mixed feeding. The respondents adopting a grazing system showed that their animals were 4.10 times (OR 4.10) more likely to have a high-level infestation as compared to those kept in a manger or mixed feeding system while the other variable in the model is held constant. The acaricides commonly used by respondents also significantly affected the tick infestation level (OR 1.77). The interaction term (i.e. sex, literacy, knowledge about TTBDs and attitude towards tick control) was not significant in this analysis (Fig. 1, Table 6).

**Table 6.** Association between socio-demographic variables of respondents and tick infestation level by R software analysis with different R packages

Coefficient	Estimate	Std. Error	*Z* value	*P* value	Odds raito
Intercept	2.33544	0.83395	2.800	0.005103**	10.33
Sex (female *vs* male)	0.39980	0.44346	0.902	0.367293	1.491
Literacy (illiterate *vs* literate)	1.25456	0.74840	−1.676	0.093676	3.51
Floor (kachha *vs* pakka)	1.64023	0.49015	3.346	0.000819***	5.16
Feeding system (grazing *vs* manger/mixed)	1.41199	0.44494	3.173	0.001506**	4.10
Knowledge about TTBDs (yes *vs* no)	−0.04726	0.81512	0.058	0.953761	0.95
Acaricides used (cypermentrin, deltamethrin, ivermectin, amitraz and mixed)	0.57355	0.25822	2.221	0.026340*	1.77
Attitude towards tick control (unfavourable *vs* favourable)	0.17804	0.58454	0.305	0.760681	1.20

The odds ratio is the ‘exponential’ of the estimate obtained in glm model (log regression). Significant at ***P < 0.001, **P < 0.01, *P < 0.05.

### Resistance status of deltamethrin and fipronil

**Figure 2.**
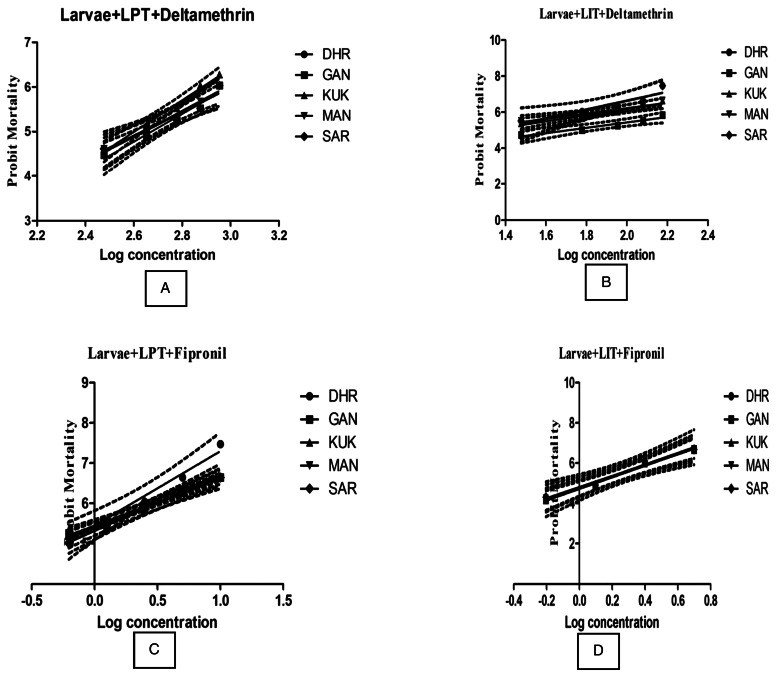
Regression curves showing probit mortality in larval packet test (LPT, A and B) and larval immersion test (LIT, C and D) against log concentration of chemical acaricides (deltamethrin and fipronil) in 5 field isolates of *Rhipicephalus microplus* from Dhar district, Madhya Pradesh, India: DHR, Dhar; GAN, Gandhwani; KUK, Kukshi; MAN, Manawar; SAR, Sardarpur.

To identify the generation of acaricide resistance in tick population collected from different sites of a district, the larval-based assays, i.e. LPT and LIT, were conducted in the laboratory against DLM and FIP and the larval mortality was recorded after 24 h. In case of *R. microplus*, the tested isolates were highly resistant to DLM (RR = 33.9–39.9) as observed by LPT (Table 7). Surprisingly, in case of LIT, a low level of resistance against DLM was detected (RF = 1.2–4.3) (Table 7). All the isolates were susceptible to FIP by LPT (RR = 0.17–0.24) and LIT (RR = 0.48–0.51). The LC50 values were ranging from 400.69 to 471.6 ppm and 15.02 to 51.42 ppm against DLM in LPT and LIT format. The lower mortality slopes were observed in all isolates as compared to reference susceptible IVRI-I strain (3.42 ± 0.49) indicating the presence of more heterogeneous DLM-resistant population of *R. microplus*. The results indicated that the ticks of this area developed resistance against DLM (Fig. 2, Table 7).

**Table 7.** Mortality slope, *R*^2^, LC_50_ with 95% CI and RR50 values of deltamethrin and fipronil against larvae of *R. microplus* by using LPT and LIT

Acaricide	LPT						LIT				
	Tick isolates	Mortality (slope ± s.e.)	*R*^2^	LC50 (95% CI)	RR50	RL	Mortality (slope ± s.e.)	*R*^2^	LC50 (95% CI)	RR50	RL
DDeltamethrin	DHR	2.769 ± 0.39	0.94	441.43 (405.06–481.06)	37.4	III	2.455 ± 0.56	0.86	21.20(19.23–23.36)	1.7	I
	GAN	3.117 ± 0.35	0.96	471.6 (436.93–509.01)	39.9	III	1.499 ± 0.24	0.92	51.42 (43.87–60.26)	4.3	I
	KUK	3.561 ± 0.22	0.98	400.69 (374.77–428.39)	33.9	III	1.740 ± 0.25	0.94	20.70 (18.05–23.73)	1.7	I
	MAN	2.904 ± 0.33	0.96	435.85 (401.5–473.08)	36.9	III	1.487 ± 0.23	0.92	15.02 (14.57–15.48)	1.2	S
	SAR	2.685 ± 0.40	0.93	428.57 (392.21–468.29)	36.3	III	2.528 ± 0.61	0.84	22.80 (20.74–25.05)	1.9	I
	IVRI-I	3.42 ± 0.49	0.87	11.8 (11.6–12.0)	1.0	S	3.42 ± 0.49	0.87	11.80 (11.6–12.0)	1.0	S
FFipronil	DHR	1.847 ± 0.19	0.96	0.56 (0.49–0.63)	0.23	S	2.804 ± 0.22	0.98	1.21 (1.11–1.31)	0.50	S
	GAN	1.222 ± 0.10	0.97	0.47 (0.38–0.57)	0.19	S	2.873 ± 0.20	0.98	1.24(1.14–1.34)	0.51	S
	KUK	1.261 ± 0.03	0.99	0.49 (0.40–0.59)	0.20	S	2.852 ± 0.24	0.98	1.22 (1.12–1.32)	0.50	S
	MAN	1.176 ± 0.05	0.99	0.42 (0.34–0.51)	0.17	S	2.865 ± 0.35	0.96	1.18 (1.08–1.28)	0.49	S
	SAR	1.391 ± 0.11	0.97	0.59 (0.49–0.69)	0.24	S	2.760 ± 0.27	0.97	1.16 (1.06–1.26)	0.48	S
	IVRI-I	7.67 ± 2.4	0.84	2.4 (2.38–2.42)	1.00	S	7.67 ± 2.4	0.84	2.4 (2.38–2.42)	1.00	S

DHA, Dhar; GAN, Gandhwani; KUK, Kukshi; MAN, Manawar; SAR, Sardarpur; IVRI-I, reference susceptible tick strain; RR50 (median), resistance ratio; RL, resistance level [susceptible (S) = RR < 1.4; level I: 1.5 < RR < 5; level II: 5.1 < RR < 25; level III: 26 < RR < 40; level IV: RR > 41].

**Figure 3.**
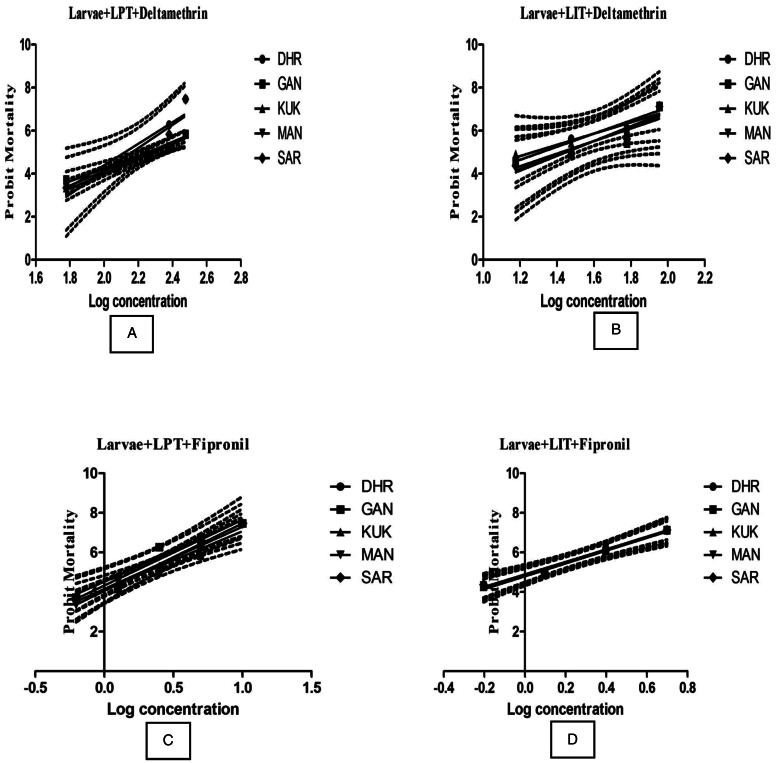
Regression curves showing probit mortality in larval packet test (LPT, A and B) and larval immersion test (LIT, C and D) against log concentration of chemical acaricides (deltamethrin and fipronil) in 5 field isolates of *Hyalomma anatolicum* from Dhar district, Madhya Pradesh, India: DHR, Dhar; GAN, Gandhwani; KUK, Kukshi; MAN, Manawar; SAR, Sardarpur.

The LPT- and LIT-based resistance data of *H. anatolicum* against DLM and FIP are documented in Table 8. Results revealed that all the field isolates were resistant to DLM at level II (RF = 11.1–16.6) by LPT. Similarly, in case of LIT, an initiation of resistance to DLM was detected (RF = 1.5–2.3). Like *R. microplus* all the isolates of *H. anatolicum* were also found susceptible to FIP. The LC50 values were in the range from 132.17 to 194.90 ppm and 18.53 to 28.04 ppm against DLM in LPT and LIT, respectively. The lower mortality slopes were seen in all the samples in comparison to IVRI-I strain (3.42 ± 0.49) except Dhar isolate (5.002 ± 1.23) indicating the presence of more heterogeneous DLM-resistant populations of *H. anatolicum* (Fig. 3).

**Table 8.** Mortality slope, *R*^2^, LC_50_ with 95% CI and RR50 values of deltamethrin and fipronil against larvae of *H. anatolicum* by using LPT and LIT

Acaricide	LPT						LIT				
	Tick isolates	Mortality (slope ± s.e.)	*R*^2^	LC50 (95% CI)	RR50	RL	Mortality (slope ± s.e.)	*R*^2^	LC50 (95% CI)	RR50	RL
DDeltamethrin	DHR	5.002 ± 1.23	0.84	132.17 (126.02–138.61)	11.1	II	3.048 ± 0.44	0.95	20.63 (19.07–22.31)	1.7	I
	GAN	2.915 ± 0.33	0.96	182.88 (168.53–198.44)	15.4	II	2.928 ± 1.09	0.78	26.46 (24.39–28.70)	2.2	I
	KUK	3.448 ± 0.30	0.97	194.90 (181.89–208.83)	16.5	II	2.616 ± 0.63	0.89	18.53 (16.91–20.29)	1.5	I
	MAN	3.379 ± 0.21	0.98	180.91 (168.60–194.11)	15.3	II	3.436 ± 0.74	0.91	28.04 (26.16–30.05)	2.3	I
	SAR	5.374 ± 1.19	0.87	145.61 (139.30–152.20)	12.3	II	3.257 ± 0.86	0.87	27.54 (25.59–29.62)	2.3	I
	IVRI-I	3.42 ± 0.49	0.87	11.8 (11.6–12.0)	1.0	S	3.42 ± 0.49	0.87	11.8 (11.6–12.0)	1.0	S
FFipronil	DHR	3.125 ± 0.35	0.96	1.41 (1.30–1.52)	0.58	S	3.144 ± 0.24	0.98	1.07 (0.99–1.15)	0.44	S
	GAN	3.320 ± 0.49	0.93	1.58 (1.47–1.69)	0.65	S	3.240 ± 0.26	0.98	1.15 (1.06–1.23)	0.47	S
	KUK	2.948 ± 0.38	0.95	1.97 (1.81–2.13)	0.82	S	3.239 ± 0.19	0.99	1.11 (1.03–1.19)	0.46	S
	MAN	3.410 ± 0.31	0.97	1.88 (1.75–2.01)	0.78	S	3.201 ± 0.26	0.98	1.09 (1.01–1.17)	045	S
	SAR	3.144 ± 0.20	0.98	1.85 (1.71–1.99)	0.77	S	3.142 ± 0.29	0.98	1.11 (1.02–1.19)	0.46	S
	IVRI-I	7.67 ± 2.4	0.84	2.4 (2.38–2.42)	1.00	S	7.67 ± 2.4	0.84	2.4 (2.38–2.42)	1.00	S

DHA, Dhar; GAN, Gandhwani; KUK, Kukshi; MAN, Manawar; SAR, Sardarpur; IVRI-I, reference susceptible tick strain; RR50 (median), resistance ratio; RL, resistance level [susceptible (S) = RR < 1.4; level I: 1.5 < RR < 5; level II: 5.1 < RR < 25; level III: 26 < RR < 40; level IV: RR > 41].

## Discussion

This research is the first to investigate the knowledge, attitudes and behaviours of livestock owners in Madhya Pradesh, India, about ticks and tick control measures. The report also covers the perceptions of stakeholders and livestock farmers in the study region about these limitations. Historically, most of the animal health researches worldwide were focused on pastoral regions (Catley *et al*., 2014; Queenan *et al*., 2017). Pastoral communities predominate in the majority of African nations; in contrast, mixed crop-livestock farming practices and production systems are widespread in India (Hemme *et al*., 2013). More than half of the surveyed respondents did not know how their livestock become infested with ticks or where ticks are typically located in the environment, despite the fact that every respondent had encountered a tick problem. According to some respondents, ticks are less prevalent in the winter. As temperature is an important factor in several tick developmental processes, including moulting, oviposition and questing, low temperature in the winter is typically expected to slow down these processes (Estrada-Peña and Fernández-Ruiz, 2020). Ticks are only known to search for hosts at temperatures over 7°C (Süss, 2008; Namgyal *et al*., 2021a). However, winter in the Dhar district of Madhya Pradesh is typically dry and cold, with a mean temperature of 15–20°C. In this part of Madhya Pradesh, ticks are more prevalent in rainy seasons due to high temperatures and humidity conditions.

The present study revealed a lack of knowledge among livestock owners on TBDs. The similarities in education and livestock-rearing techniques suggest that these findings may be applicable to other areas and countries (Chakraborty *et al*., 2023). Ticks and TBDs lead to extensive veterinary and public health issues, particularly in India. TBDs and severe tick infestations have been linked to reduced milk, meat and other animal product output in several developing nations, along with increased animal sickness and death. Ticks transmit a greater number of illnesses than any other blood-feeding arthropod globally, posing a threat to people, their pets and cattle (Rehman *et al*., 2019; Ngnindji-Youdje *et al*., 2022). Indigenous cow breeds are often believed to have high resistance to ticks and may be reared without proper attention on tick management (Minjauw and McLeod, 2003; Phanchung *et al*., 2007; Jonsson *et al*., 2014).

Face-to-face interviews in the present study helped to understand farmers' knowledge, their attitudes towards TBDs and acaricide application patterns in fields. The current data indicate majority of farmers to be illiterate and lacking awareness of the TBDs. Most of the farmers used traditional uncemented sheds to maintain their livestock. Besides, the majority of animal owners were using chemical acaricides on their animals without adhering to suitable tick management methods and dosage regime. Animals were classified as having low, moderate or high levels of tick infestation based on the presence of 25, 100 and 150 ticks, as documented by Chigure *et al*. (2018). We observed that several farms were severely infected with ticks, leading to a decrease in total productivity. A high negative association was seen between the frequency of acaricide usage and the proportion of tick-infested animals. This suggests that the frequent and effective use of acaricides is a significant factor contributing to the variation in tick prevalence across various farms. Indian researchers determined DLM as the most commonly used acaricide in the field, followed by cypermethrin, amitraz and ivermectin and observed that farms experienced high tick infestation, possibly due to owners' lack of awareness about the correct use of acaricides and the resistance of ticks to the products being used (Ghosh *et al*., 2015; Chigure *et al*., 2018; Shakya *et al*., 2020; Upadhaya *et al*., 2020). According to Hussain *et al*. (2021), out of the livestock owners in the survey, 51 (45.5%) used acaricides frequently, but 49 (43.8%) did not have appropriate disposal methods for spent acaricidal bottles and unused goods, opting to dispose of them in general waste streams, including farm drainage systems. Thirty-four livestock owners, accounting for 30.4% of the total, did not use any acaricides in the year before to our visit, although they had used them previously. Regarding application techniques, 26 farmers (23.2%) used systemic acaricide, while 34 farmers (30.4%) employed topical treatments for tick control. Tesfaye and Abate (2023) noted that the respondents estimated the amount of the acaricide instead of monitoring doses (whether sprayed or injected) before treatment. In our findings, researchers conducted an investigation that revealed that native breeds were allowed to graze outside, but cross-bred animals were kept confined in a shed. Native breeds have a lower tick infection rate compared to cross-breeds. In many countries that are developing, herd owners acquire acaricide use information from persons without expertise, leading to improper acaricide practices. In the present surveyed places, rural veterinary stores and shop workers with little technical knowledge serve as the primary source of information for farmers, leading to inadequate and improper acaricidal practices. In a prior research conducted in Kenya (Mugambi *et al*., 2012), it was shown that many herd owners get information on acaricide administration from untrained vet shop attendants. This lack of sufficient training in animal health care might result in herd owners engaging in harmful practices. Recommendations to farmers were given to rotate the use of acaricides in cattle to reduce acaricide resistance and for cost-effective treatment due to the high frequency of TTBDs (Ghosh and Azhahianambi, 2007).

Most respondents and farmers lack awareness of TBDs and expressed unfavourable attitudes about tick management during face-to-face interviews. Similarly, researchers worldwide shared their views on the knowledge and attitudes of respondents. For instance, Lontsi-Demano *et al*. (2021) conducted a cross-sectional survey to evaluate farmers' knowledge and practices regarding ticks and the management of TBDs. They found that herd managers possessed a fundamental understanding of ticks and their impact on animals. Namgyal *et al*. (2021b) observed that 128 out of 246 respondents (52%) had sufficient information regarding ticks as carriers of illnesses in people and animals. Hussain *et al*. (2021) studied how cattle producers perceive and handle tick infestation. In another study, Hussain *et al*. (2021) determined that 47.3% of cattle owners were knowledgeable about TBDs and used sandy flooring, indicating awareness of the related risk factors. In the present study, the most popular animal feeding system is mixed type (57.5%) followed by stall feeding (37.5%). Similarly, Hussain *et al*. (2021) noted that 25% of farmers used stall feeding, and 53.6% embraced both methods. Tesfaye and Abate (2023) reported that the prevalence of tick infestation sometimes increased. Insufficient grazing habitat has caused animal herds to cluster in some areas, resulting in a higher spread of tick infestation.

The present study used the LPT, initially developed by Stone and Haydock (1962), and the LIT, developed by Shaw (1966), to identify and monitor resistance to acaricides. In this study, we found that, all 5 isolates of *R. microplus* and *H. anatolicum* collected from 5 different sub-divisions of Dhar district were found to be resistant to DLM which may be due to extensive use of synthetic pyrethroid compounds and easy availability of this compound. Accordingly, the DLM resistance in both the tick species has been reported across the country. For example, Jyothimol *et al*. (2014) reported comparatively low level of resistance in field tick larvae collected from 2 districts of Kerala. Shyma *et al*. (2015) and Gaur *et al*. (2016) also reported DLM resistance in field ticks collected from Haryana, Rajasthan and Gujarat states of India. Similarly, Kumar *et al*. (2017) reported tick larvae from 6 districts of Andhra Pradesh state and reported RF of 1.05–8.78. The ineffectiveness of DLM was also reported from the states like Uttar Pradesh, Assam and Maharashtra at resistance levels I–IV (Chigure *et al*., 2018; Upadhaya *et al*., 2020; Khating *et al*., 2024). DLM resistance has also been reported in *R. microplus* from West Africa (Adehan *et al*., 2016; Yessinou *et al*., 2018), Mexico (Rosario-Cruz *et al*., 2009) and Australia (Gurrero *et al*., 2012). Besides *R. microplus*, Becker *et al*. (2019) reported resistance in *R. sanguineus* isolate collected from 8 Porto Alegre metropolitan areas, Brazil with RF 1.18–5.67.

Earlier, country-specific discriminating concentration (DC = 2 × LC_99_) of FIP was determined as 9.6 ppm using LPT against reference susceptible IVRI-I strain of *R. microplus* (Kumar *et al*., 2016) for differentiating between susceptible and resistant ticks. In the present study, all the collected isolates of *R. microplus* and *H. anatolicum* were found susceptible to FIP. This may be due to the high cost and comparatively less use of FIP for tick control. Recently, Shakya *et al*. (2020) characterized 25 isolates collected from 6 states (Madhya Pradesh, Uttarakhand, Meghalaya, Assam, Gujarat and Haryana) and reported RF in the range of 0.39–10.9. Analysing the data, it is observed that FIP is not widely adopted in most of the countries for the management of ticks, and therefore, reports on the development of FIP resistance in tick population are not frequently available in the literature.

## Conclusion

The results provide useful insights to aid in the development of educational and outreach programmes that may go beyond the research region. The proper knowledge of TBDs among the animal owners is essential for effective management of tick infestation and improvement of animal health and productivity. The present study to mitigate acaricide resistance and TBDs revealed significant gaps in awareness and proper management strategies. Some farmers showed a basic understanding of tick control, the majority lacked comprehensive knowledge of acaricide resistance and effective disease prevention. Future research should focus on developing targeted educational programmes to enhance farmers' knowledge and attitudes towards sustainable tick control practices. Further, studies should explore other alternatives to chemical acaricides, to minimize acaricide resistance and TBDs in livestock.

## Data Availability

Supplementary data may be provided on request to corresponding author.
